# How serotonin shapes moral judgment and behavior

**DOI:** 10.1111/nyas.12229

**Published:** 2013-09-24

**Authors:** Jenifer Z Siegel, Molly J Crockett

**Affiliations:** 1 Wellcome Trust Centre for Neuroimaging, University College LondonLondon, United Kingdom; 2 Laboratory for Social and Neural Systems Research, University of ZürichZürich, Switzerland

**Keywords:** serotonin, moral judgment, harm aversion, fairness

## Abstract

Neuroscientists are now discovering how hormones and brain chemicals shape social behavior, opening potential avenues for pharmacological manipulation of ethical values. Here, we review recent studies showing how altering brain chemistry can alter moral judgment and behavior, focusing in particular on the neuromodulator serotonin and its role in shaping values related to harm and fairness. We synthesize previous findings and consider the potential mechanisms through which serotonin could increase the aversion to harming others. We present a process model whereby serotonin influences social behavior by shifting social preferences in the positive direction, enhancing the value people place on others’ outcomes. This model may explain previous findings relating serotonin function to prosocial behavior, and makes new predictions regarding how serotonin may influence the neural computation of value in social contexts.

## Introduction

How does the brain produce moral behavior? What biological mechanisms determine whether a given individual harms or helps others? And can our neurobiology be manipulated for good? Of late, questions like these have arisen at the interface of neuroscience, ethics, and the law. Here, we examine the contributions of a single brain chemical—serotonin—to moral judgment and behavior.

Serotonin is a monoamine neurotransmitter that is evolutionarily ancient and well preserved across mammals. It is one of the most widely distributed neurochemicals in the mammalian nervous system, making its precise functions difficult to pinpoint; however, serotonin is more concentrated in certain structures than others. Anatomical studies illustrate the highest densities of serotonin concentrations in various limbic structures, such as the cingulate, entorhinal, insular, and temporopolar regions, along with the ventral and pallidal regions of the striatum^1^ and the medial orbitofrontal cortex.^2^ Notably, this set of regions bears a striking resemblance to the so-called social brain^3^—those regions supporting social cognition and decision making.

Not surprisingly, then, serotonin has long been implicated in social behavior across species.^4^,^5^ For example, polymorphisms in the serotonin transporter gene have been linked to personality traits related to aggression, neuroticism, and impulsivity.^6^–^11^,^30^ In both primates and humans, serotonin function tends to covary positively with prosocial behaviors such as grooming, cooperation, and affiliation, and tends to covary negatively with antisocial behaviors such as aggression and social isolation.^12^–^19^ Such prosocial and antisocial behaviors are likely precursors to human morality.^20^–^22^

Despite abundant evidence linking serotonin to morally relevant social behaviors, the neurobiological and psychological mechanisms mediating these relationships remain unclear. A recent meta-analysis encompassing 175 independent samples and over 6,500 total participants found a reliable inverse relationship between serotonin and aggression, but failed to identify the specific factors explaining the heterogeneity in study outcomes.^23^ One challenge facing research in this area is the complexity of both moral behavior and the serotonin system itself. Understanding how serotonin modulates moral behavior thus requires precise behavioral tools for measuring aspects of moral behavior, combined with targeted pharmacological tools for manipulating serotonin in the brain.

Moral codes dictate how people should treat one another, and most of these focus on two facets of social relationships. The first prescribes caring for others and prohibits harm; the second relates to the fair distribution of resources and reciprocity in social interactions.^24^,^25^ Concerns for harm and fairness play a central role in moral codes across cultures,^26^ and there is some evidence that these building blocks of morality shape social behavior in primates.^27^–^29^

In the following, we present evidence that serotonin modulates human concerns for harm and fairness, and we examine the potential mechanisms. We first consider how serotonin influences harm aversion in moral judgment and aversive processing more generally, and then examine how serotonin shapes behavioral responses to fairness and reciprocity. Finally, we synthesize these findings into a theoretical process model whereby serotonin influences social behavior by shifting social preferences in the positive direction, enhancing the value people place on others’ outcomes. This is not a comprehensive review; instead, we focus specifically on studies conducted in humans employing controlled, experimental manipulations of the serotonin system in the laboratory. For a recent comprehensive review of serotonin and social behavior, we refer readers to Kiser *et al.;*^5^ for a discussion of genetic polymorphisms of the serotonin system and their relation to social cognition, see Skuse.^30^

## Harm aversion and morality

Violence toward others is restrained by a seemingly deep-rooted aversion to harmful actions.^31^,^32^ Such *harm aversion* infuses moral judgments;^33^ people tend to judge harming an innocent person as forbidden, even when doing so would ultimately achieve a greater good.^34^,^35^ Harm aversion appears to shape judgments in moral dilemmas, in which people must judge whether it is morally permissible to harm one person in order to save many others. One classic set of dilemmas involves a runaway trolley that is heading down the tracks toward five workers, who will die if you do nothing. In one variant of the problem (“switch”), you have access to a switch that will divert the trolley onto a different set of tracks, where there is a single worker. If you flip the switch, the single worker will die, but the five others will be saved. In another variant (“push”), you and a large man are standing on a footbridge over the tracks. You can push the large man off the footbridge and onto the tracks, where his body will stop the trolley before it hits the five workers. Although the switch and push variants are matched with reference to outcomes, people are much less likely to judge it morally permissible to push the man than to flip the switch.^34^–^36^

Why do people react so differently to the two scenarios? One influential hypothesis posits that personal cases like push elicit stronger emotional responses than do impersonal cases like switch, and these emotional reactions drive harm-averse judgments in the former to a larger extent than in the latter.^37^ Incidental negative emotions like disgust increase the likelihood of harm-averse judgments, even when the emotions are unrelated to the dilemmas under consideration.^38^,^39^ Neuroimaging studies have demonstrated that harm-averse moral judgments engage brain regions previously implicated in emotional processing.^34^,^40^–^42^ Further evidence for the relationship between emotional responses to harm and moral judgment comes from a recent report that physiological reactivity to witnessing fake violent acts against people (vs. violent acts against objects) predicted moral judgments; those people who showed the strongest physiological reaction to witnessing violent acts were the least likely to endorse harming one to save many others.^32^

Note that most studies of moral judgment ask participants whether it is morally permissible to actively cause harm, for example, “Is it morally permissible to push the man?” In these studies, negative cues associated with the harmful action in question could trigger a withdrawal reflex, making subjects less likely to endorse active responses. We might expect this Pavlovian aversive withdrawal process to be particularly strong in personal scenarios that often involve lurid descriptions of the violent actions. There is some evidence supporting this hypothesis. Increasing the vividness of the descriptions of harm in moral dilemmas reduces endorsement of harmful actions.^43^ Ugazio *et al*.^39^ found that disgust, a withdrawal-related negative emotion, reduced subjects’ endorsement of harmful actions, whereas anger, an approach-related negative emotion, had the opposite effect. Finally, Pastötter *et al*.^44^ recently reported that negative emotions reduced the endorsement of harming one to save many when subjects were asked explicitly whether harming one was morally permissible. However, negative emotions increased the endorsement of harming one to save many when subjects were asked explicitly whether not harming one was morally permissible. In other words, negative emotions increased the likelihood that subjects would say “no, that is not permissible,” regardless of the question asked. These findings support the notion that aversive states promote behavioral withdrawal, which can translate into harm-averse moral judgments when those judgments are framed in relation to action permissibility.

## Serotonin and harm aversion

It turns out that serotonin has been implicated in precisely this aspect of aversive processing—namely behavioral withdrawal in the presence of aversive cues. Early studies in rats demonstrated that global brain serotonin depletion made them insensitive to punishment.^45^ In humans, serotonin levels are positively correlated with harm-avoidant personality traits,^46^,^47^ and psychiatric disorders involving aversive processing, such as anxiety and depression, are associated with serotonergic abnormalities. The precise motivational processes driving these findings have been subject to a long debate that has yet to be fully resolved.^48^–^54^ However, recent attempts have made considerable progress, both by integrating previous theories of serotonin function and by extending the logic from existing computational models of dopamine.^50^,^53^,^54^

One current hypothesis is that serotonin plays a key role at the intersection of aversion and inhibition. Under normal conditions, the presence of aversive outcomes leads to behavioral inhibition, which can manifest in a reduced probability of action or in slowed response times. Modest depletion of brain serotonin in humans abolishes this aversively motivated behavioral inhibition,^4^,^55^,^56^ suggesting that serotonin is important for promoting behavioral suppression or withdrawal in the face of aversive predictions. Note that behavioral inhibition in response to aversive outcomes reflects at least two concurrent processes: instrumental aversive predictions linking actions to outcomes and Pavlovian aversive predictions linking stimuli and contexts to outcomes. Recent data suggest that serotonin mediates reactions to the latter, Pavlovian process; serotonin depletion abolished inhibition of responses in the presence of aversive stimuli, regardless of whether the responses themselves led to punishment.^56^

Serotonin's involvement in these rather basic aspects of aversive processing suggests that its influence could translate upward into effects on moral judgment. There is some evidence pointing in this direction; neuroimaging studies of moral judgment have shown that imagining harmful acts against others engages brain regions with dense serotonergic projections, including the anterior cingulate cortex, ventromedial prefrontal cortex (vmPFC), amygdala, and striatum.^34^,^40^,^42^,^57^ Moreover, patients with damage to the vmPFC show impaired harm aversion in moral judgment, in the sense that they are more likely to endorse harming one person to save many others.^58^,^59^ If serotonin plays a key role in harm aversion, could enhancing serotonin function produce effects on moral judgment opposite to those observed in the vmPFC patients?

**Figure 1:**
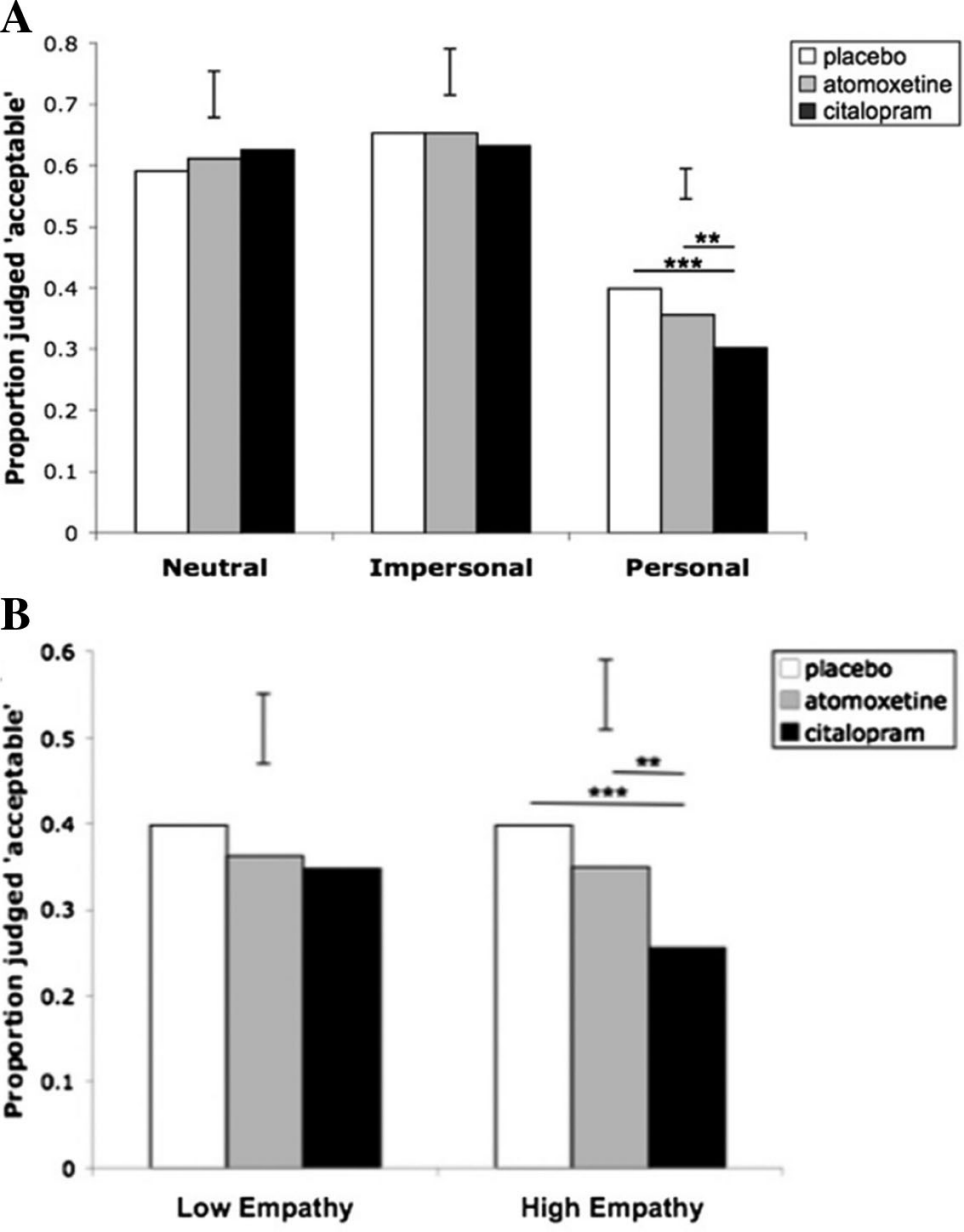
Serotonin shapes moral judgments. (A) Relative to placebo and the noradrenaline reuptake inhibitor atomoxetine, the serotonin reuptake inhibitor citalopram made subjects less likely to endorse harming one person to save many others, specifically when harms were emotionally salient. (B) The effects of citalopram on moral judgdment were strongest in subjects high in empathy. Figures adapted from Ref. 60.

Crockett *et al*.^60^ tested this hypothesis by investigating the effects of the selective serotonin reuptake inhibitor (SSRI) citalopram on moral judgments in a set of moral dilemmas. Citalopram enhances serotonin function by blocking its reuptake into the presynaptic terminal following release, thus prolonging its actions in the synapse. The set of dilemmas included personal and impersonal variants similar to the push and switch cases described above. The effects of citalopram were contrasted with those of atomoxetine, a noradrenaline reuptake inhibitor, and placebo in a double-blind within-subjects study. Relative to both atomoxetine and placebo, citalopram made people less likely to endorse harming one to save many others. In other words, citalopram enhanced harm aversion in moral judgment^60^ (Fig. 1A).

Crockett *et al*. further examined whether individual differences in empathy moderated the effects of the drug. On the basis of their scores on the Interpersonal Reactivity Index,^61^ subjects were split into high- and low-empathy groups. Subjects with lower empathy scores showed almost no effect of citalopram on moral judgment; the effect of the drug in the group overall was driven almost entirely by subjects with higher empathy scores, who showed a strong effect of citalopram on judgment (Fig. 1B). Subjects high in empathy may possess higher levels of harm aversion at baseline, which were further boosted by citalopram.

Notably, the effects of citalopram on moral judgment were specific to personal dilemmas involving emotionally salient harms. Recall the data suggesting that serotonin is critical for translating aversive Pavlovian cues into behavioral inhibition.^56^ If a similar process unfolds as subjects ponder moral dilemmas, then enhancing serotonin function could boost subjects’ responsiveness to the aversive Pavlovian cues present in the descriptions of the dilemmas, making them more averse to the suggested harmful action and more likely to disapprove of it. The results described in Crockett *et al*.^60^ are consistent with this proposal.

Blair^31^ proposed that human aggression is constrained by a “violence inhibition mechanism” that initiates a withdrawal response when activated by distress cues. We suggest that a similar mechanism operates for imagined harms in the case of moral judgment, which could account for serotonin's parallel effects in inhibiting actual harms (in the case of aggression) and imagined harms (in the case of moral judgment).

## Serotonin, fairness, and reciprocity

Humans are often selfish, but also care about the interests of others—for example, people are sometimes willing to incur costs to achieve fair outcomes, punish unkind behavior, and reward kind behavior.^62^ Such social preferences may have played an important role in human evolution, as they could maximize one's fitness in social contexts.^62^–^65^ Preferences for positive reciprocity (repaying kindness with kindness) motivate cooperation in social dilemmas that pit personal profit against social welfare. Preferences for negative reciprocity motivate costly punishment of those who violate social norms. Preferences for fairness motivate actions that seek to establish equitable outcomes. Here, we review evidence that serotonin modulates social preferences across these domains.

Cooperative behaviors in social dilemmas have been linked to serotonin function. One study found that after 2 weeks’ treatment with citalopram, participants were significantly less likely to behave in a self-interested manner in a modified version of the prisoner's dilemma that allowed participants to act selfishly, cooperatively, or charitably.^66^ Modest depletion of brain serotonin levels produced the opposite effect on cooperation in the prisoner's dilemma.^67^ These findings suggest that serotonin function is related to positive social preferences, that is, the positive valuation of others’ outcomes. However, one disadvantage of social dilemmas as measures of social preferences is their complexity. Preferences for positive reciprocity undoubtedly motivate cooperation in the prisoner's dilemma, but cooperative behavior is also sensitive to other factors, most notably subjects’ beliefs about whether their partner is likely to cooperate—subjects are more likely to cooperate if they believe their partner will also cooperate.^62^ These studies thus cannot establish whether serotonin modulates social preferences themselves, or alternatively, the beliefs upon which the preferences are predicated.

Preferences for negative reciprocity and fairness have been extensively studied using the ultimatum game (UG). The UG consists of two players, a proposer and a responder, who must agree on a way to share a sum of money, or neither will receive anything. The proposer must offer a division of the sum to the responder, who must make a decision to either accept or reject this proposal. If the responder accepts the offer, both players are paid; if he rejects, neither is paid. Perfectly selfish responders will accept any nonzero offer, but responders with preferences for fairness or reciprocity will reject offers perceived to be unfair—typically less than about 30% of the stake.^68^ Rejecting an unfair offer satisfies fairness goals because the resulting outcome—zero for both players—is perfectly equitable. Rejecting an unfair offer satisfies preferences for negative reciprocity because it punishes the proposer, depriving him of a larger amount.

Several studies have investigated the relationship between serotonin function and responder behavior in the UG. Emmanuele *et al*.^69^ reported that platelet serotonin levels were inversely correlated with responders’ rejection rates. However, given that serotonin does not penetrate the blood–brain barrier, plasma levels may not correspond to central serotonin levels. A more recent study found that the density of serotonin transporters in the dorsal raphe nucleus—a proxy measure for serotonin function—was inversely correlated with responders’ rejection rates.^70^ Although these studies suggest an association between serotonin and preferences for fairness and reciprocity, pharmacological manipulations have furthered these claims with causal evidence.

Crockett *et al*.^71^ examined the effects of reducing serotonin availability on responders’ behavior in the UG. Responders were more likely to reject unfair offers following depletion of central serotonin, relative to placebo.^71^ A subsequent study tested whether enhancing serotonin function with the SSRI citalopram would produce the opposite effect on rejection behavior. Relative to both placebo and the noradrenaline reuptake inhibitor atomoxetine, citalopram reduced responders’ rejection rates in the UG.^72^ In both studies, the behavioral changes resulting from the serotonin manipulations could not be attributed to changes in mood, the ability to inhibit motor responses, or judgments about the fairness of the offers—suggesting that the manipulations affected behavior by directly altering social preferences.

**Figure 2:**
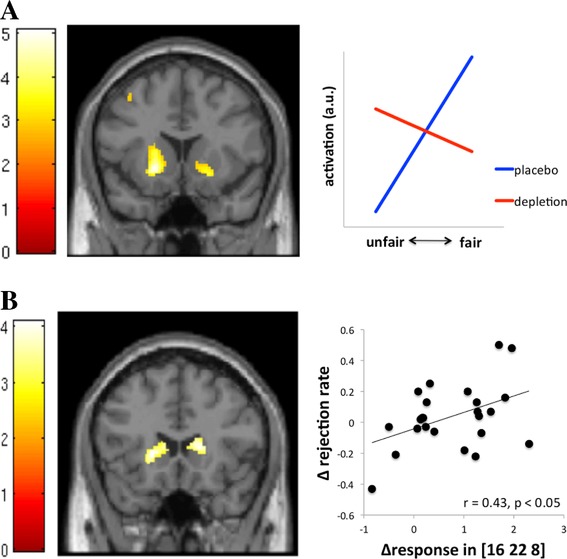
Serotonin shapes social preferences in the striatum. (A) Relative to placebo, serotonin depletion blunted responses in the ventral striatum during the receipt of fair offers in the ultimatum game (UG). (B) Relative to placebo, serotonin depletion enhanced responses in the dorsal striatum during the rejection of unfair offers in the UG. Individual differences in the effects of depletion on dorsal striatal activity were positively correlated with individual differences in the effects of depletion on rejection behavior. Figures adapted from Ref. 73.

Rejection of unfair offers in the UG can be explained by either preferences for fairness or preferences for reciprocity. Crockett *et al*.^73^ combined pharmacological manipulations with functional neuroimaging to investigate how serotonin modulates each of these preferences separately. Previous neuroimaging studies demonstrated that fair social exchanges stimulate activity in the ventral striatum and medial PFC,^74^–^76^ suggesting that activity in these regions reflects preferences for fairness. Crockett *et al*.^73^ examined the effects of serotonin depletion on ventral striatal and medial PFC responses to the receipt of fair offers in the UG, and found that serotonin depletion blunted these regions’ responses to fairness (Fig. 2A). Serotonin levels, therefore, appear to positively correlate with (neural) preferences for fairness.

Meanwhile, negative reciprocity has been associated with activation in the dorsal striatum. Neuroimaging studies found activation in the dorsal striatum during retaliatory actions following both reception and observations of unfair behaviors.^77^,^78^ This activity was only observed for effective punishment; symbolic reciprocal actions that did not reduce the norm violator's payoff did not stimulate activity in the dorsal striatum.^78^ Furthermore, the magnitude of dorsal striatal activity was correlated with the amount the subject was willing to pay to punish the violator. Collectively, these findings suggest that the dorsal striatum signals the instrumental value of negative reciprocity, consistent with its broader role in goal-directed reward processing.^79^ Crockett *et al*.^73^ demonstrated that serotonin depletion enhanced responses in the dorsal striatum during rejection of unfair offers in the UG, relative to placebo. This effect was specific to trials in which subjects actively rejected the unfair offers, relative to trials in which subjects simply received unfair offers but did not have the opportunity to reject. Moreover, the effects of the serotonin manipulation on dorsal striatal activity were positively correlated with the effects of the serotonin manipulation on rejection behaviors (Fig. 2B). These results suggest that the dorsal striatum plays a causal role in negative reciprocity, and that serotonin levels are negatively correlated with neural and behavioral preferences for negative reciprocity.

**Figure 3:**
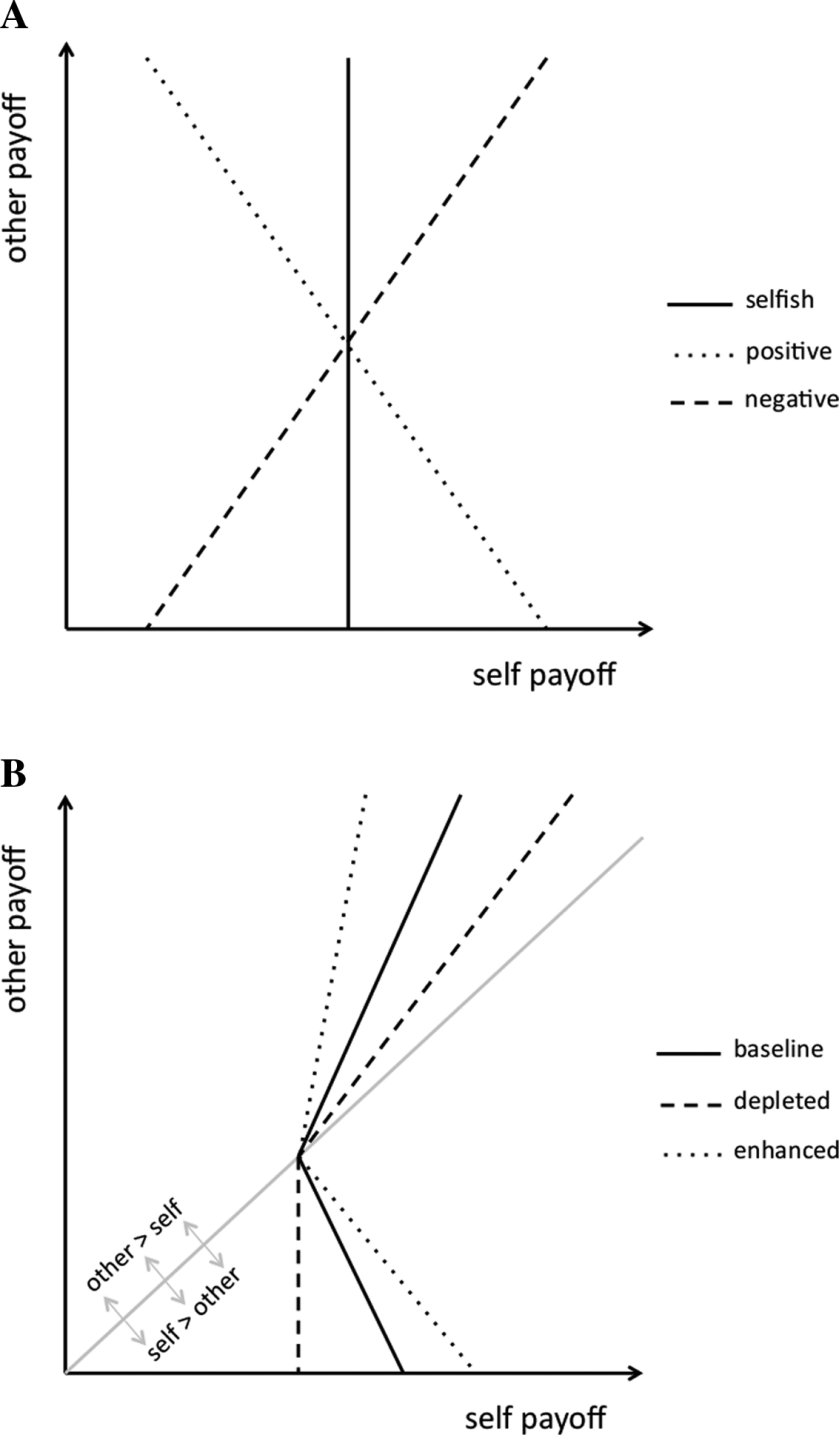
How serotonin shapes social preferences. (A) Theoretical social preference profiles, represented as indifference curves. All points on the curve have equivalent subjective value. Pure selfishness (solid line) is represented by a vertical indifference curve; for a given self-payoff, others’ payoffs do not affect utility. Positive social preferences (dotted line) are represented by a downward-sloping indifference curve; altruists are willing to sacrifice some of their own payoff to benefit others. Negative social preferences (dashed line) are represented by an upward-sloping indifference curve; spiteful individuals are willing to sacrifice some of their own payoff to reduce the payoffs of others. (B) Theoretical social preference profiles representing inequality aversion and its hypothesized modulation by serotonin. Inequality-averse individuals display downward-sloping positive social preferences when they are in an advantageous position (to the right of the gray equality line), and upward-sloping negative social preferences when they are in a disadvantageous position (to the left of the gray equality line). We propose that serotonin shifts social preferences in the positive (counterclockwise) direction.

The association between serotonin and social reward processing dovetails with previous reports linking serotonin function to the processing of non-social rewards^80^,^99^,^100^ and recent studies showing that social and monetary rewards engage overlapping regions of the striatum.^81^,^82^ Collectively, these findings indicate that the role of serotonin in value computation goes beyond a simple enhancement or inhibition of reward processing in general; instead, serotonin's effects appear to depend on the social context. We suggest that serotonin amplifies neural representations of positive social preferences, whereas serotonin depletion shifts neural value computations toward selfish or even negative social preferences. This perspective is consistent with earlier behavioral research indicating a positive correlation between serotonin function and prosocial behaviors, but goes a step further by proposing how serotonin affects the preferences that drive those behaviors (Fig. 3).

## Synthesis: serotonin and social preferences?

Our synthesis of experimental findings advocates a causal role for serotonin in both harm aversion and social preferences for fairness and reciprocity. Could these two ostensibly distinct facets of morality instead reflect a single underlying dimension? More specifically, can harm aversion be thought of as a type of positive social preference?

Figure 3A illustrates three theoretical social preference profiles, represented as indifference curves (where all points on the curve have equivalent subjective value). An examination of Figure 3A demonstrates how harm aversion can be thought of as a type of positive social preference. Individuals with positive social preferences show downward sloping indifference curves, which means that harm to others results in utility losses for the self. As the indifference curve rotates in the clockwise direction, preferences move toward pure selfishness. Once the slope of the curve is positive, we can see that the individual in question displays negative social preferences—in which harm to others results in utility gains for the self. Counterclockwise rotations of the indifference curve thus result in increased harm aversion, whereas clockwise rotations of the indifference curve result in decreased harm aversion.

Often the social context determines whether social preferences are positive or negative: one notable example is inequality aversion.^83^ Inequality-averse individuals (Fig. 3B) show positive social preferences when they are in an advantageous position (to the right of the gray line denoting equal payoffs), and negative social preferences when they are in a disadvantageous position (to the left of the gray line denoting equal payoffs). The indifference curves in Figure 3B capture most people's behavior in the UG.^68^

We suggest that serotonin shifts the slope of the indifference curve in the direction of positive social preferences. Crockett *et al*.^71^,^73^ demonstrated that serotonin depletion amplifies negative social preferences under conditions of disadvantageous inequality (Fig. 3B, dashed line), whereas serotonin enhancement diminishes negative social preferences in this setting (Fig. 3B, dotted line). Our model can also account for previous studies of serotonin's influence on cooperation. Assuming that people are predisposed to cooperate in social dilemmas^65^ (i.e., that their indifference curves are downward-sloping, absent concerns about inequality), serotonin augmentation should shift preferences further counterclockwise, making people more cooperative,^84^ whereas serotonin depletion should shift preferences clockwise, making people less cooperative.^67^ Finally, previous studies have shown that serotonin manipulations influence aggressive behavior, particularly in people predisposed to aggression. Assuming that aggressive individuals have upward-sloping indifference curves (i.e., they are motivated to harm others) serotonin augmentation again should shift preferences counterclockwise, reducing aggression,^85^ whereas serotonin depletion should shift preferences further clockwise, increasing aggression.^86^–^91^

Our model is primarily descriptive at the behavioral level, but makes predictions that can be tested at the neural level. For example, value-processing regions such as the ventral striatum and the medial PFC show a pattern of activation consistent with inequality aversion;^76^ we predict that serotonin manipulations would alter responses in these regions as illustrated in Figure 3B (for preliminary evidence, see Ref. 73).

Finally, it is worth mentioning that social cognitive and emotional processes concerning the representation of others’ mental states and emotions (i.e., mentalizing and empathy) clearly play a role in shaping concerns for harm and fairness.^92^,^93^ Recent studies have shown that the structure and function of brain regions involved in mentalizing, such as the temporoparietal junction (TPJ) and the superior temporal sulcus, can predict positive social preferences and subsequent generosity.^94^,^95^ Similarly, regions associated with empathy, such as the anterior insula and anterior cingulate cortex, are sensitive to the moral status of others^96^ and are correlated with altruistic helping.^97^,^98^ A still-open question, therefore, is whether serotonin modulates moral behavior indirectly by affecting empathic representations in the TPJ, insula, and anterior cingulate cortex, or directly by altering the neural computations of social preferences in the striatum and the medial PFC. Although initial evidence supports the latter view,^73^ further work is needed in this area to understand how individual differences in empathy moderate the effects of serotonin on moral judgment and behavior.^72^

## Concluding remarks

Research into the neural basis of moral judgment and behavior has exploded over the past decade. The vast majority of this work has involved neuroimaging; these studies have provided valuable insights into the neural correlates of moral decisions, but are correlational in nature. More recent studies employing intervention methods, such as pharmacological manipulations and brain stimulation, have provided additional information about the brain systems that are causally involved in moral decisions. Future work employing these techniques will benefit from specified theoretical frameworks that generate novel predictions about how targeted interventions will modify moral judgment and behavior.

## References

[1] Varnäs K, Halldin C, Hall H (2004). Autoradiographic distribution of serotonin transporters and receptor subtypes in human brain. Hum. Brain Mapp.

[2] Way BM (2007). Architectonic distribution of the serotonin transporter within the orbitofrontal cortex of the vervet monkey. Neuroscience.

[3] Sanfey AG (2007). Social decision-making: insights from game theory and neuroscience. Science.

[4] Crockett MJ, Clark L, Robbins TW (2009). Reconciling the role of serotonin in behavioral inhibition and aversion: acute tryptophan depletion abolishes punishment-induced inhibition in humans. J. Neurosci.

[5] Kiser D (2012). The reciprocal interaction between serotonin and social behaviour. Neurosci. Biobehav. Rev.

[6] Retz W (2004). Association of serotonin transporter promoter gene polymorphism with violence: relation with personality disorders, impulsivity, and childhood ADHD psychopathology. Behav. Sci. Law.

[7] Greenberg BD (2000). Association between the serotonin transporter promoter polymorphism and personality traits in a primarily female population sample. Am. J. Med. Genet.

[8] Holmes A (2003). Mice lacking the serotonin transporter exhibit 5-HT1A receptor-mediated abnormalities in tests for anxiety-like behavior. Neuropsychopharmacology.

[9] Lesch KP (1996). Association of anxiety-related traits with a polymorphism in the serotonin transporter gene regulatory region. Science.

[10] Mazzanti CM (1998). Role of the serotonin transporter promoter polymorphism in anxiety-related traits. Arch. Gen. Psychiatr.

[11] Nielsen DA (1994). Suicidality and 5-hydroxyindoleacetic acid concentration associated with a tryptophan hydroxylase polymorphism. Arch. Gen. Psychiatr.

[12] Doudet D (1995). Cerebral glucose metabolism, CSF 5-HIAA levels, and aggressive behavior in rhesus monkeys. Am. J. Psychiatr.

[13] Higley J (1996). Stability of interindividual differences in serotonin function and its relationship to severe aggression and competent social behavior in rhesus macaque females. Neuropsychopharmacology.

[14] Higley JD (1992). Cerebrospinal fluid monoamine and adrenal correlates of aggression in free-ranging rhesus monkeys. Arch. Gen. Psychiatr.

[15] Raleigh M (1980). Serotonergic influences on the social behavior of vervet monkeys (*Cercopithecus aethiops sabaeus*. Exp. Neurol.

[16] Linnoila M (1983). Low cerebrospinal fluid 5-hydroxyindoleacetic acid concentration differentiates impulsive from nonimpulsive violent behavior. Life Sci.

[17] Krakowski M (2003). Violence and serotonin: influence of impulse control, affect regulation, and social functioning. J. Neuropsychiatr. Clin. Neurosci.

[18] Moskowitz D (2001). The effect of tryptophan on social interaction in everyday life-a placebo-controlled study. Neuropsychopharmacology.

[19] Knutson B (1998). Selective alteration of personality and social behavior by serotonergic intervention. Am. J. Psychiatr.

[20] Brosnan SF, de Waal FB (2012). Fairness in animals: where to from here. Soc. Just. Res.

[21] Proctor D (2013). Chimpanzees play the ultimatum game. Proc, Natl. Acad. Sci.

[22] Stevens JR, Cushman FA, Hauser MD (2005). Evolving the psychological mechanisms for cooperation. Annu. Rev. Ecol. Evol. Syst.

[23] Duke AA (2013). Revisiting the serotonin—aggression relation in humans: a meta-analysis. Psychol. Bull.

[24] Haidt J (2007). The new synthesis in moral psychology. Science.

[25] Graham J, Haidt J (2010). Beyond beliefs: religions bind individuals into moral communities. Pers. Soc. Psychol. Rev.

[26] Rochat P (2009). Fairness in distributive justice by 3-and 5-year-olds across seven cultures. J. Cross-Cult. Psychol.

[27] de Waal F (2010). Prosocial primates: selfish and unselfish motivations. Philos. Trans. R. Soc. B: Biol. Sci.

[28] Brosnan SF, Schiff HC, de Waal FB (2005). Tolerance for inequity may increase with social closeness in chimpanzees. Proc. R. Soc. B: Biol. Sci.

[29] de Waal FB, Leimgruber K, Greenberg AR (2008). Giving is self-rewarding for monkeys. Proc. Natl. Acad. Sci.

[30] Skuse D (2006). Genetic influences on the neural basis of social cognition. Philos. Trans. R. Soc. B: Biol. Sci.

[31] Blair RJR (1995). A cognitive developmental approach to morality: investigating the psychopath. Cognition.

[32] Cushman F, Young L (2011). Patterns of moral judgment derive from nonmoral psychological representations. Cogn. Sci.

[33] Haidt J (2001). The emotional dog and its rational tail: a social intuitionist approach to moral judgment. Psychol. Rev.

[34] Greene JD (2001). An fMRI investigation of emotional engagement in moral judgment. Science.

[35] Cushman F, Young L, Hauser M (2006). The role of conscious reasoning and intuition in moral judgment testing three principles of harm. Psychol. Sci.

[36] Hauser M (2007). A dissociation between moral judgments and justifications. Mind Lang.

[37] Greene JD (2007). The Secret Joke of Kant's Soul.

[38] Schnall S (2008). Disgust as embodied moral judgment. Pers. Soc. Psychol. Bull.

[39] Ugazio G, Lamm C, Singer T (2012). The role of emotion for moral judgments depends on the type of emotion and moral scenario. Emotion.

[40] Greene JD (2004). The neural bases of cognitive conflict and control in moral judgment. Neuron.

[41] Schaich Borg J (2006). Consequences, action, and intention as factors in moral judgments: an fMRI investigation. J. Cogn. Neurosci.

[42] Shenhav A, Greene JD (2010). Moral judgments recruit domain-general valuation mechanisms to integrate representations of probability and magnitude. Neuron.

[43] Bartels DM (2008). Principled moral sentiment and the flexibility of moral judgment and decision making. Cognition.

[44] Pastötter B (2013). To push or not to push? Affective influences on moral judgment depend on decision frame. Cognition.

[45] Tye N, Everitt B, Iversen SD (1977). 5-Hydroxytryptamine and punishment. Nature.

[46] Gerra G (2000). Neuroendocrine correlates of temperamental traits in humans. Psychoneuroendocrinology.

[47] Hansenne M, Ansseau M (1999). Harm avoidance and serotonin. Biol. Psychol.

[48] Brodie BB, Shore PA (1957). A concept for a role of serotonin and norepinephrine as chemical mediators in the brain. Ann. NY. Acad. Sci.

[49] Deakin J (1983). Roles of serotonergic systems in escape, avoidance and other behaviours. Theory Psychopharmacol.

[50] Cools R, Nakamura K, Daw ND (2010). Serotonin and dopamine: unifying affective, activational, and decision functions. Neuropsychopharmacology.

[51] Soubrie P (1986). Reconciling the role of central serotonin neurons in human and animal behavior. Behav. Brain Sci.

[52] Deakin JW, Graeff FG (1991). 5-HT and mechanisms of defence. J. Psychopharmacol.

[53] Dayan P, Huys QJ (2009). Serotonin in affective control. Annu. Rev. Neurosci.

[54] Boureau Y-L, Dayan P (2010). Opponency revisited: competition and cooperation between dopamine and serotonin. Neuropsychopharmacology.

[55] Crockett M (2011). Converging evidence for central 5-HT effects in acute tryptophan depletion. Mol. Psychiatr.

[56] Crockett MJ (2012). Serotonin modulates the effects of pavlovian aversive predictions on response vigor. Neuropsychopharmacology.

[57] Kédia G (2008). An agent harms a victim: a functional magnetic resonance imaging study on specific moral emotions. J. Cogn. Neurosci.

[58] Koenigs M (2007). Damage to the prefrontal cortex increases utilitarian moral judgements. Nature.

[59] Ciaramelli E (2007). Selective deficit in personal moral judgment following damage to ventromedial prefrontal cortex. Soc. Cogn. Affect. Neurosci.

[60] Crockett MJ (2010). Serotonin selectively influences moral judgment and behavior through effects on harm aversion. Proc. Natl. Acad. Sci.

[61] Davis MH, Luce C, Kraus SJ (1994). The heritability of characteristics associated with dispositional empathy. J. Pers.

[62] Fehr E, Fischbacher U, Gächter S (2002). Strong reciprocity, human cooperation, and the enforcement of social norms. Hum. Nat.

[63] Gächter S, Renner E, Sefton M (2008). The long-run benefits of punishment. Science.

[64] Andreoni J, Miller J (2003). Giving according to GARP: an experimental test of the consistency of preferences for altruism. Econometrica.

[65] Rand DG (2009). Positive interactions promote public cooperation. Science.

[66] Tse WS, Bond AJ (2002). Difference in serotonergic and noradrenergic regulation of human social behaviours. Psychopharmacology.

[67] Wood RM (2006). Effects of tryptophan depletion on the performance of an iterated Prisoner's Dilemma game in healthy adults. Neuropsychopharmacology.

[68] Camerer CF (2003). Strategizing in the brain. Science.

[69] Emanuele E (2008). Relationship between platelet serotonin content and rejections of unfair offers in the ultimatum game. Neurosci. Lett.

[70] Takahashi H (2012). Honesty mediates the relationship between serotonin and reaction to unfairness. Proc. Natl. Acad. Sci.

[71] Crockett MJ (2008). Serotonin modulates behavioral reactions to unfairness. Science.

[72] Crockett MJ (2010). Impulsive choice and altruistic punishment are correlated and increase in tandem with serotonin depletion. Emotion.

[73] Crockett MJ (2013). Serotonin modulates striatal responses to fairness and retaliation in humans. J. Neurosci.

[74] Tabibnia G, Satpute AB, Lieberman MD (2008). The sunny side of fairness preference for fairness activates reward circuitry (and disregarding unfairness activates self-control circuitry). Psychol. Sci.

[75] Zaki J, Mitchell JP (2011). Equitable decision making is associated with neural markers of intrinsic value. Proc. Natl. Acad. Sci.

[76] Tricomi E (2010). Neural evidence for inequality-averse social preferences. Nature.

[77] Strobel A (2011). Beyond revenge: neural and genetic bases of altruistic punishment. Neuroimage.

[78] Quervain DJF (2004). The neural basis of altruistic punishment. Science.

[79] O'Doherty J (2004). Dissociable roles of ventral and dorsal striatum in instrumental conditioning. Science.

[80] Ranade SP, Mainen ZF (2009). Transient firing of dorsal raphe neurons encodes diverse and specific sensory, motor, and reward events. J. Neurophysiol.

[81] Izuma K, Saito DN, Sadato N (2008). Processing of social and monetary rewards in the human striatum. Neuron.

[82] Rademacher L (2010). Dissociation of neural networks for anticipation and consumption of monetary and social rewards. Neuroimage.

[83] Fehr E, Schmidt KM (1999). A theory of fairness, competition, and cooperation. Q. J. Eco.

[84] Tse WS, Bond AJ (2003). Consequences of displaying abnormal social behaviour: avoidance and reduction of social reinforcement. J. Affect. Dis.

[85] Berman ME (2009). Serotonin augmentation reduces response to attack in aggressive individuals. Psychol. Sci.

[86] Gibbons JL (1979). Manipulations of dietary tryptophan: effects on mouse killing and brain serotonin in the rat. Brain Res.

[87] Cleare A, Bond A (1995). The effect of tryptophan depletion and enhancement on subjective and behavioural aggression in normal male subjects. Psychopharmacology.

[88] Bjork JM (1999). The effects of tryptophan depletion and loading on laboratory aggression in men: time course and a food-restricted control. Psychopharmacology.

[89] Moeller F (1996). Tryptophan depletion and aggressive responding in healthy males. Psychopharmacology.

[90] Bjork JM (2000). Differential behavioral effects of plasma tryptophan depletion and loading in aggressive and nonaggressive men. Neuropsychopharmacology.

[91] Dougherty DM (1999). Influence of trait hostility on tryptophan depletion-induced laboratory aggression. Psychiatr. Res.

[92] Gray K, Young L, Waytz A (2012). Mind perception is the essence of morality. Psychological Inquiry.

[93] Gleichgerrcht E, Young L (2013). Low levels of empathic concern predict utilitarian moral judgment. PLoS One.

[94] Morishima Y (2012). Linking brain structure and activation in temporoparietal junction to explain the neurobiology of human altruism. Neuron.

[95] Hare RD, Neumann CS (2010). The role of antisociality in the psychopathy construct: comment on Skeem and Cooke (2010). Psychol. Assess.

[96] Singer T (2006). Empathic neural responses are modulated by the perceived fairness of others. Nature.

[97] FeldmanHall O (2012). Differential neural circuitry and self-interest in real vs hypothetical moral decisions. Soc. Cogn. Affect. Neurosci.

[98] Hein G (2010). Neural responses to ingroup and outgroup members’ suffering predict individual differences in costly helping. Neuron.

[99] McCabe C, Mishor Z, Cowen PJ, Harmer CJ (2010). Diminished neural pro-cessing of aversive and rewarding stimuli during selective serotonin re-uptake inhibitor treatment. Biol. Psychiatr.

[100] Seymour B, Daw ND, Roiser JP (2012). Serotonin selectively modulates reward value in human decision-making. J. Neurosci.

